# Cytoarchitectonic parcellation and functional characterization of four new areas in the caudal parahippocampal cortex

**DOI:** 10.1007/s00429-021-02441-2

**Published:** 2022-01-06

**Authors:** Sophie Stenger, Sebastian Bludau, Hartmut Mohlberg, Katrin Amunts

**Affiliations:** 1 Cécile and Oskar Vogt-Institute for Brain Research, University Hospital Düsseldorf, Medical Faculty, Heinrich-Heine-University Düsseldorf, Düsseldorf, Germany; 2 Institute of Neuroscience and Medicine 1 (INM-1), Research Centre Jülich, Jülich, Germany

**Keywords:** Cytoarchitecture, Human brain mapping, Parahippocampal gyrus, Collateral sulcus, Parahippocampal place area, Julich-Brain

## Abstract

Brain areas at the parahippocampal gyrus of the temporal–occipital transition region are involved in different functions including processing visual–spatial information and episodic memory. Results of neuroimaging experiments have revealed a differentiated functional parcellation of this region, but its microstructural correlates are less well understood. Here we provide probability maps of four new cytoarchitectonic areas, Ph1, Ph2, Ph3 and CoS1 at the parahippocampal gyrus and collateral sulcus. Areas have been identified based on an observer-independent mapping of serial, cell-body stained histological sections of ten human postmortem brains. They have been registered to two standard reference spaces, and superimposed to capture intersubject variability. The comparison of the maps with functional imaging data illustrates the different involvement of the new areas in a variety of functions. Maps are available as part of Julich-Brain atlas and can be used as anatomical references for future studies to better understand relationships between structure and function of the caudal parahippocampal cortex.

**Supplementary Information:**

The online version contains supplementary material available at 10.1007/s00429-021-02441-2.

## Introduction

The caudal parahippocampal cortex (PHC) is part of the ventral temporal cortex and its transition to the occipital cortex. It has been activated in studies targeting different tasks of visual–spatial processing as well as in memory (Aminoff et al. 2013; Bohbot et al. 2015). Visual functions linked to the PHC include map reading, spatial orientation, navigation and spatial memory (Epstein and Kanwisher 1998; Aguirre et al. 1996; Janzen et al. 2007; Maguire et al. 1998; Aguirre and D'Esposito 1999; Mellet et al. 2000; Baumann and Mattingley 2021). For example, the posterior portion of the PHC reacts strongly to stimuli that show places and scenes, which has earned it the name “parahippocampal place area” (PPA) (Epstein and Kanwisher 1998; Weiner et al. 2018, 2017). Recently, a functional region of interest (ROI) map of early visual and category-selective regions in human ventral and lateral occipito-temporal cortex was published (Rosenke et al. 2021). The map showed, among other areas, the location of the PPA as well as the fusiform face areas (FFA-1/2; Pinsk et al. 2009), which were located within the region of the collateral sulcus and the parahippocampal gyrus. On the other hand, the PHC is involved in the formation of episodic memory, e.g., associative memory (Davachi et al. 2003; Kirwan and Stark 2004; Tendolkar et al. 2008; Düzel et al. 2003; Henke et al. 1999; Hales et al. 2009; Yang et al. 2008). Associative memory refers to links between items (e.g., “snow” and “winter”), which are basic building blocks of episodic memory and allows to integrate associations between people, places, actions, emotions, noises, smells, etc. into an overall construct. In this context, the PHC has been shown to be involved in the retrieval of contextual information (Eichenbaum et al. 2007; Diana et al. 2007). The memory of names and their corresponding faces is also incumbent on the PHC (Kirwan and Stark 2004). In addition, this region seems to be also involved in the processing of emotional stimuli (Smith et al. 2004; Gosselin et al. 2006; Mitterschiffthaler et al. 2007; Van den Stock et al. 2014), and the processing of auditory (Gosselin et al. 2006; Mitterschiffthaler et al. 2007; Arnott et al. 2008; Engelien et al. 2006) and odor stimuli (Kjelvik et al. 2012; Cerf-Ducastel and Murphy 2009).

The PHC is connected with a variety of regions that are responsible for processing visual information and creating memory. This includes afferents from visually and somatosensory associated regions as well as connections to the retrosplenial cortex, the parietal lobe and cingulate cortex (Kim and Kim 2005; Caspers et al. 2011; Libby et al. 2012; Rushworth et al. 2006). In addition, the PHC has connections to the medial temporal lobe. Strong connections have also been found between the PHC and the hippocampus (Libby et al. 2012).

Anatomically, the lateral border of the parahippocampal gyrus is formed by the collateral sulcus, which is followed in anterior direction by the rhinal sulcus. The parahippocampal gyrus is located medially to the fusiform gyrus. The hippocampal fissure limits it medially. Rostrally, the parahippocampal gyrus merges into the uncus, while caudally, it becomes divided by the anterior calcarine fissure with the isthmus of the retrosplenial cortex dorsally, and the lingual gyrus ventrally (Duvernoy et al. 1991; Ono et al. 1990).

**Fig. 1:**
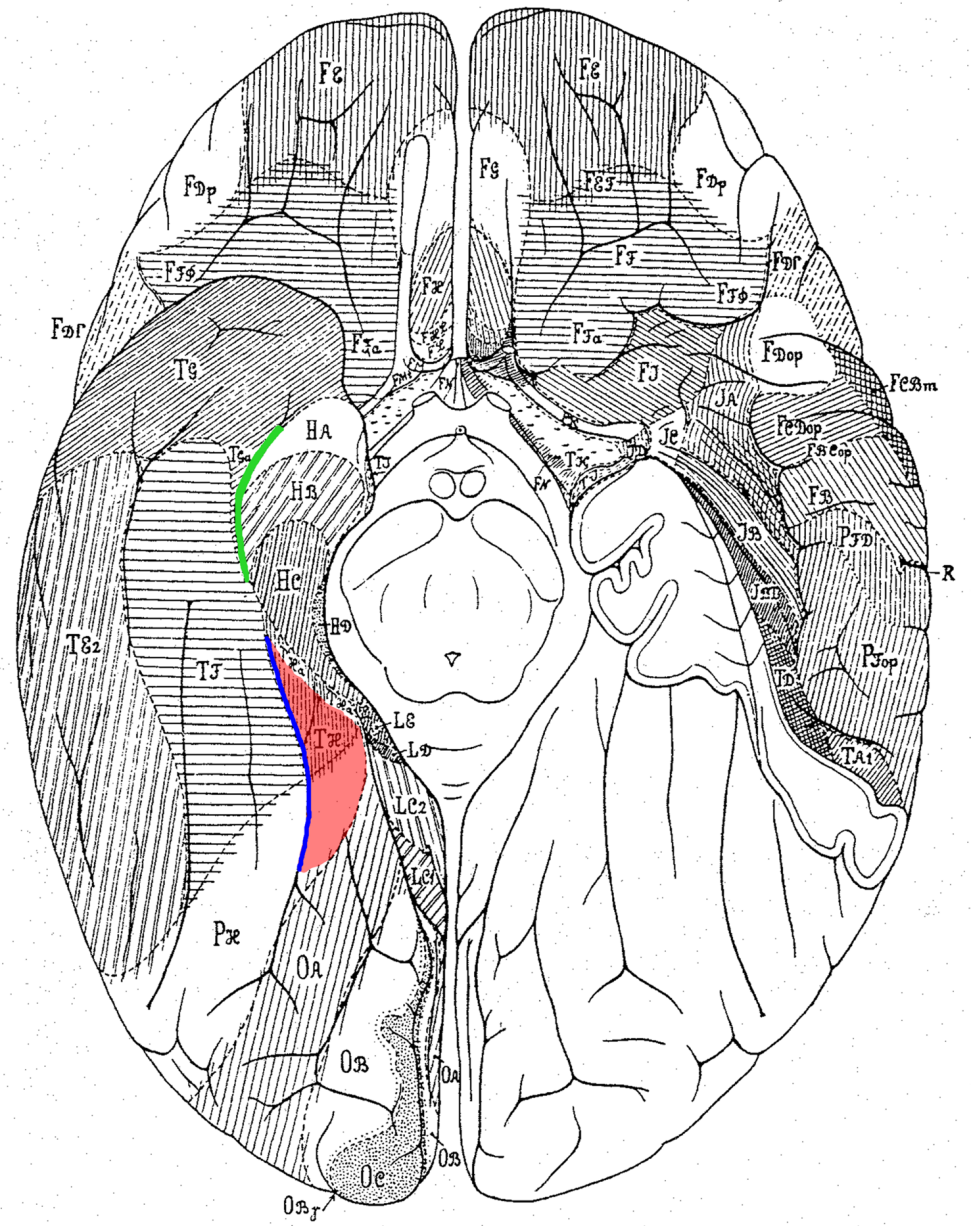
Historical cytoarchitectonic map of von Economo and Koskinas (1925), basal view. The region of interest is marked in red. It includes areas PH and TH. The collateral sulcus is marked in blue, the rhinal sulcus is marked in green

The microstructural correlates of the functional spectrum in which the PHC is involved, is less well understood. The PHC roughly corresponds to the caudal part of Brodmann’s area 28 (1909) and/or areas TH and PH according to the map of von Economo and Koskinas (1925) (Fig. 1). According to more recent literature, cytoarchitectonic areas of the fusiform gyrus (FG1-4; Lorenz et al. 2017; Caspers et al. 2013) as identified by our group using an observer-independent mapping approach in ten postmortem brains are laterally and caudally adjacent. Probabilistic cytoarchitectonic maps of the four fusiform areas have been applied as an anatomical reference for interpreting activations from in vivo neuroimaging (Rosenke et al. 2016; Weiner et al. 2017). For example, Gomez et al. (2017) used these maps to better distinguish place- and face selective areas, and to describe their specific changes during ontogeny.

Along the same line of reasoning, we, therefore, addressed the question of the microstructural segregation of the adjacent parahippocampal gyrus and collateral sulcus, to provide cytoarchitectonic correlates for the different functions associated with this region. For this purpose, we examined histological sections of ten postmortem brains, identified and mapped four new areas Ph1, Ph2, Ph3 and CoS1, computed probabilistic maps in stereotaxic space, compared the maps with findings from functional neuroimaging studies, and provided them as a freely available resource and tool as part of the Julich-Brain atlas.

## Materials and methods

### Histological processing of postmortem brains

**Fig. 2:**
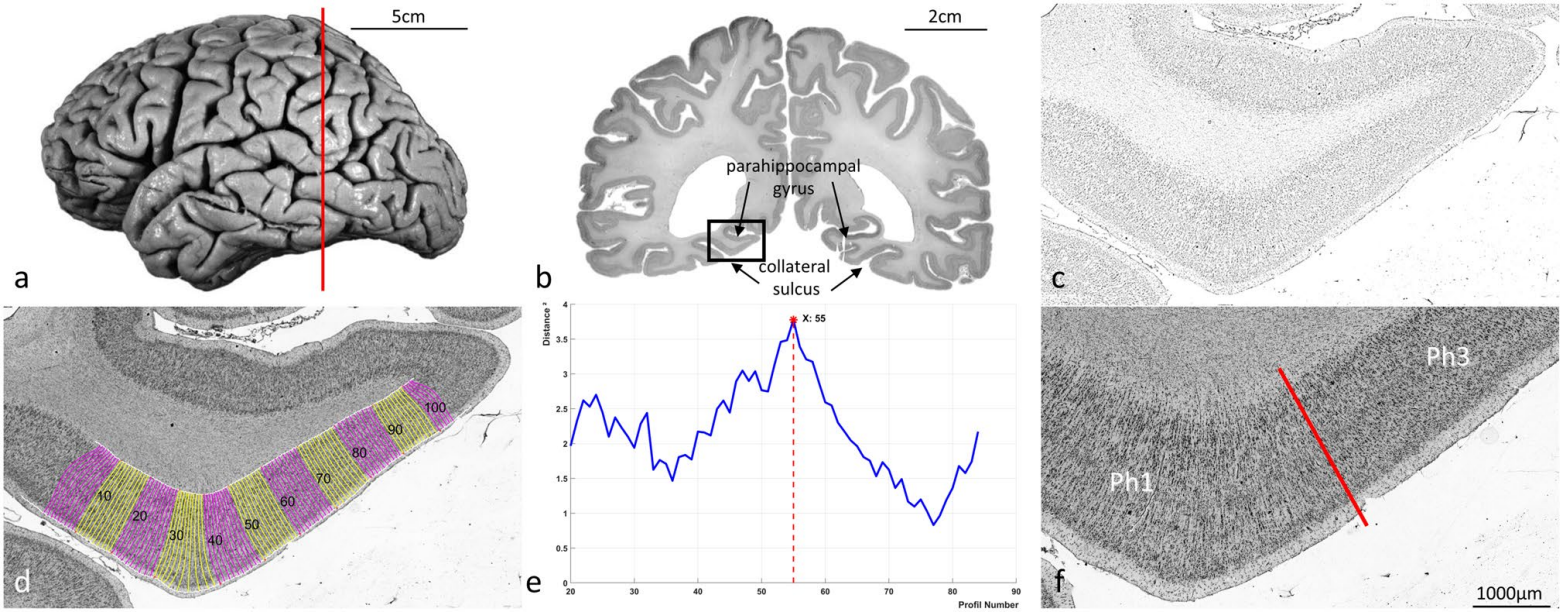
Observer-independent identification of cytoarchitectonic borders. **a** Postmortem brain number 4 (Table 1). The sectioning plane is marked in red. **b** Coronal section stained for cell bodies with ROI (black rectangle). This ROI was digitized and subsequently transformed into a GLI image. **c** Inverted GLI image. **d** Equidistant traverses were calculated and GLI profiles were extracted along these traverses. **e** Mahalanobis distances as a function on the position of the profile along the cortical ribbon (example for a block size of 20 profiles); significant maximum at profile 55. **f** Projection of the identified border between areas Ph1 and Ph3 to the original image

The cytoarchitecture was analysed in a sample of ten human postmortem brains (five male, and five female) obtained through the body donor program of the Anatomical Institute of the University of Düsseldorf (ethics approval of the medical faculty of the Heinrich-Heine-University Düsseldorf #4863). Postmortem brain number 4 is shown in Fig. 2a. The handedness of the donors was unknown. There were no psychiatric or neurological diseases in the medical records (Table 1). Braincode 20 corresponded to the BigBrain 1, a microscopical brain model at 20 μm isotropic resolution (Amunts et al. 2013). The procedure of histological processing and analysis of images was described in detail previously (Amunts et al. 2020). In short, the brains were removed within a postmortem delay of 8–24 h. They were fixed in 4% formalin or Bodian’s fixative for 6 months or more. MR scans of the fixed brains were taken to obtain undistorted reference images for subsequent 3D-reconstruction, and to correct for artefacts introduced during histological processing. For this purpose, a *T*1-weighted 3D-FLASH sequence (flip angle 40°, repetition time = 40 ms, and echo time = 5 ms), acquired by a Siemens 1.5 T scanner (Erlangen, Germany), was used. Afterwards, the brains were embedded in paraffin and serially cut in the coronal plane into sections of 20 μm (Fig. 2b). Every 15th section was mounted on a glass slide, and stained for cell bodies using a modified silver-staining technique (Merker 1983; Uylings et al. 1999). For the BigBrain, each section was processed and 3D-reconstructed. The subsequent cytoarchitectonic analysis was based on examination of every fourth stained section, i.e., every 60th section of the series. This resulted in a distance of 1.2 mm between the investigated sections.

**Table 1:** List of postmortem brains that were used for cytoarchitectonic analysis

Braincode	Gender	Age (years)	Cause of death	Fresh weight (g)
1	F	79	Carcinoma of the bladder	1350
2	M	55	Rectal carcinoma	1270
4	M	75	Necrotizing glomerulonephritis	1349
6	M	54	Cardiac infarction	1622
7	M	37	Acute right heart failure/cardiac arrest	1437
8	F	72	Renal failure/renal arrest	1216
9	F	79	Cardiorespiratory insufficiency	1110
10	F	85	Mesenteric infarction	1046
12	F	43	Cardiorespiratory insufficiency	1198
20	M	65	Cardiorespiratory insufficiency	1392

Braincode 20 corresponds to the BigBrain 1 (Amunts et al. 2013). *F* female, *M* male

### Detection of cortical borders based on a multi-variate distance measure of cytoarchitectonic differences

The definition of borders was based on image analysis and statistical tools, to identify significant changes in the laminar pattern, i.e., cytoarchitecture (Schleicher et al. 2009). Histological sections of eight of ten brains were available as images with 1 μm/pixel resolution (⁓8 Gb per image, 8bit). For scanning the sections, a high throughput bright field microscope was used (TissueScope LE120, Huron Digital Pathology). The images were accessible via the Section Tracer Online Tool (Amunts et al. 2020). Rectangular regions of interest (ROI) were defined in the images of the scanned sections (Fig. 2b) and extracted with 1 μm/pixel resolution. For the remaining two brains, ROIs were defined and digitized using a light microscope with a scanning stage (Axioplan 2 imaging, ZEISS, Germany). Scanning was supported by the Zeiss image analysis software Axiovision (version 4.6), resulting in images with an in-plane resolution of 1.02 μm per pixel. Each ROI included the parahippocampal gyrus and adjacent cortex.

Digitized ROIs were converted into grey level index (GLI) images (Schleicher et al. 2009) using a Matlab based script (The MathWorks, Inc., Natick, MA, USA). The 8bit grey values of the GLI images were a measure of the volume fraction of cell bodies (Wree et al. 1982) in a square measuring field of 16 × 16 μm^2^ in the original high resolution images (Bludau et al. 2014; Schleicher et al. 2009) (Fig. 2c).

Subsequently, equidistant traverses were calculated between the contour lines labelling the border between layers I and II (outer contour line) and the layer VI/the white matter border (inner contour line; Fig. 2d). GLI profiles along these traverses were extracted perpendicular to the cortical layers. Their shape shows the changes in the GLI from the surface of the cortex to the white matter, and thus characterizes the cytoarchitecture. Each profile was normalized to a cortical depth of 100% to allow comparison of cortices with different thickness. Ten descriptive mathematical features (the mean GLI, the standard deviation, the position of the center of gravity on the profile, skewness as well as kurtosis, and the corresponding parameters of the profile’s first derivative) were used to parametrize the shape of GLI profiles and thus the underlying cytoarchitecture. These ten features were combined into a feature vector (Schleicher et al. 1999). Differences between feature vectors of adjacent blocks of GLI profiles were quantified using the Mahalanobis distance, a multivariate distance measure (Mahalanobis et al. 1949). GLI profiles were grouped into blocks of 10–24 GLI profiles (Schleicher et al. 2009; Bludau et al. 2014), and the Mahalanobis distance was calculated for each block size. A sliding window procedure was used to compute the distance for every localisation along the cortical ribbon. Local maxima of Mahalanobis distances indicated areal borders (Fig. 2e). The significance of the borders was examined using a Hotelling’s *T*2 test with Bonferroni correction for multiple comparisons (*p* < 0.001). Finally, each border was controlled by visual inspection of the histological images (Fig. 2f). Borders were accepted when they had to be consistently detected at the same positions across several block sizes and the positions had to be found at comparable positions in at least three adjacent sections.

### Analysis of volumes, surface areas and cortical thicknesses

The volumes *V* of the analysed areas were calculated as the product of the distance *s* between the investigated sections (number of sections), the thickness *T* of a histological section (20 μm), the width *x* as well as the height *y* of a pixel both measuring 0.02116 mm, the areal surface *ΣNi* over all sections (in pixels) and the shrinkage factor *F* of each individual brain using the following formula (Amunts et al. 2007; Bludau et al. 2014):

$$V=s*T*x*y*\sum Ni*F$$

The volumes were individually corrected for shrinkage caused by histological processing (Amunts et al. 2007). To compare volumes of areas from brains which differ in size, they were normalized using the individual whole brain volumes (Bludau et al. 2014). Comparisons of the relative volumes in terms of hemispherical and gender differences were made using a pairwise permutation test and a Matlab tool (The MathWorks, Inc., Natick, MA, USA) (Bludau et al. 2014). To verify the null-hypothesis that no differences exist, a Monte Carlo simulation with a repetition of one million iterations was used: for a million times each hemisphere was randomly assigned to one of two groups, male or female, or right or left hemispheres calculating the differences again and again. To consider differences in sex or hemispheres as significant they had to exceed 95% of the values under the null-hypothesis (*p* < 0.05).

The data set of the maximum probability map, described below, was projected (Operto et al. 2008) onto a published FreeSurfer version of the “Colin27” reference brain and was used to compute the surface area and the mean cortical thickness of each cytoarchitectonic area employing the FreeSurfer framework (Fischl and Dale 2000). The surface area was calculated as the mean value between the pial and smooth white matter surface, whereas the cortical thickness was computed as the mean distance value over all vertices of the triangulated areal surface patch.

### Cluster analysis

The four new areas Ph1, Ph2, Ph3 and CoS1 as well as areas FG1, FG2, FG3 and FG4 from the neighbouring fusiform gyrus (Caspers et al. 2013; Lorenz et al. 2017) were examined for similarities and differences in their cytoarchitecture based on mean GLI profiles. For this purpose, three sections with an average of 15 profiles per hemisphere were extracted for each area and brain, i.e., about 45 profiles per hemisphere (a total of 900 profiles). Care was taken to select profiles from regions, which were free of histological artifacts, large vessels or tangentially cut. Feature vectors of the GLI profiles were calculated as input for a hierarchical cluster analysis, based on the Euclidean distance and the Ward linkage procedures (Ward 1963) using Matlab (The MathWorks, Inc., Natick, MA, USA). The Euclidean distance served as a measure of cytoarchitectonic similarity: the more similar the cytoarchitectonic pattern of two areas, the smaller the Euclidean distance between their feature vectors. The results were visualized using a dendrogram.

### Computation of probability maps in stereotaxic space

Borders of areas were labelled over their full extent in both hemispheres in images of histological sections using the in-house software “Section Tracer Online Tool”, and the 3D reconstructed. The individual maps were aligned to “Colin27”, the *T*1-weighted single-subject template brain of the Montreal Neurological Institute (MNI), and the nonlinear asymmetric MNI152 2009c template space (Evans et al. 2012). After alignment to the templates, a superimposition of the individual maps was computed, resulting in a probability maps in each stereotaxic space (Amunts et al. 2020). The probabilistic maps then indicate, for each voxel of the reference brain, the probability with which an area could be found at this position. These maps also provide a measure of the intersubject variability—the higher the probability, the smaller intersubject differences between brains with respect to these areas, and vice versa. The probabilities were visualized using a colour coding from dark blue (low probability) to red (high probability).

In a next step, a maximum probability map (MPM) was created. For this purpose, each voxel was assigned to the area that had the highest probability in this particular position (Eickhoff et al. 2005, 2006; Amunts et al. 2020). Subsequently, the centers of gravity were computed. All maps of the areas can be accessed via the Julich-Brain Cytoarchitectonic Atlas (Amunts et al. 2020) and the Human Brain Atlas of the HBP as part of EBRAINS (https://ebrains.eu/service/human-brain-atlas).

The maps were then compared with coordinates of functional studies activating the parahippocampal gyrus and the collateral sulcus. Therefore, surfaces of the maximum probability maps were computed, and superimposed with reported coordinates of functional studies (Aguirre et al. 1996; Epstein et al. 1999; Hales et al. 2009; Henke et al. 1999; Janzen et al. 2007; Kirwan and Stark 2004; Kveraga et al. 2011; Sommer et al. 2005; Maguire et al. 1998). The described coordinates of the functional studies were converted into native MNI coordinates and displayed together with the new areas in the nonlinear asymmetric MNI152 2009c reference space. A published FreeSurfer surface of the most probable location of the PPA (Weiner et al. 2018) was also visualized within the MNI152 reference brain. The PPA surface representation was transferred from the published FreeSurfer fsaverage brain to a corresponding volume in the MNI152 template (Wu et al. 2018), to allow a combined visualization.

## Results

**Fig. 3:**
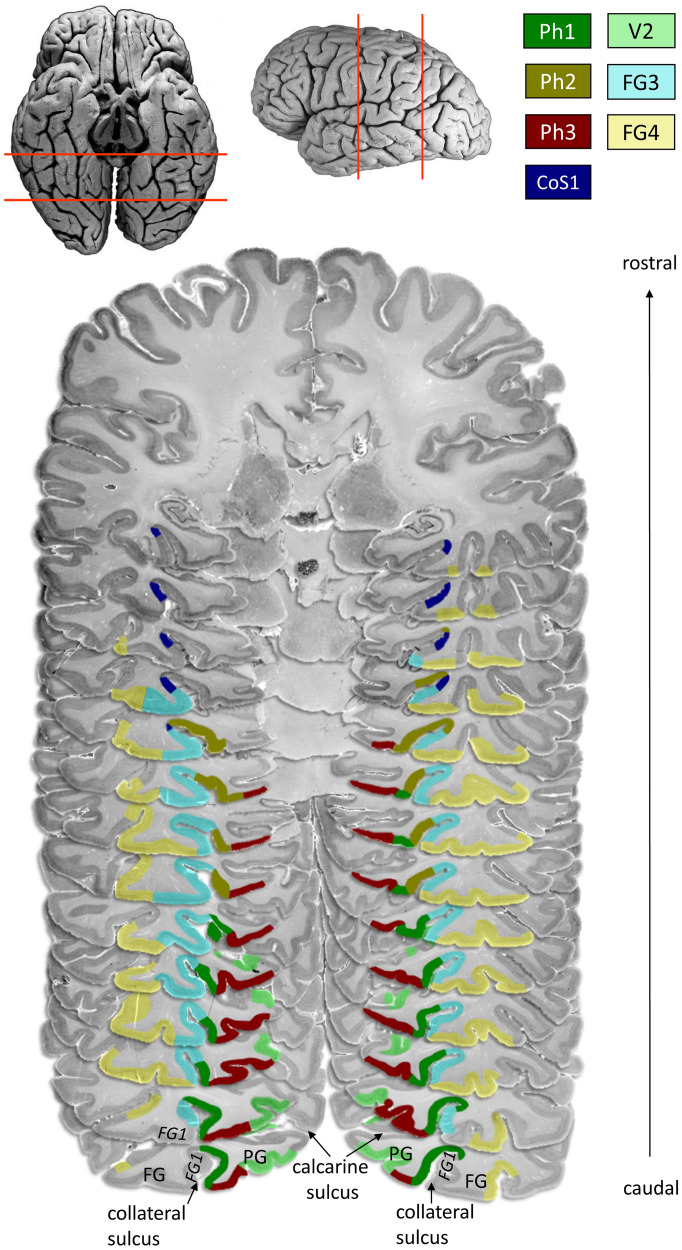
Caudal-to-rostral extent of areas Ph1, Ph2, Ph3 and CoS1 in 14 serial histological sections of postmortem brain number 1. Distance between most caudal and most rostral sections: 33 mm. Adjacent areas V2 (BA18; Amunts et al. 2000), FG1 (Caspers et al. 2013), FG3 and FG4 (Lorenz et al. 2017) are indicated. Collateral sulcus, calcarine sulcus, fusiform gyrus (FG) and parahippocampal gyrus (PG) are also labelled. The red lines in the lateral and ventral view of the postmortem brain in the upper left mark the approximate region from which the section series originates

Four new areas were identified: Ph1, Ph2, Ph3 and CoS1. Area CoS1 occupied the lateral bank of the collateral sulcus and was the most anteriorly located area in this group. It never reached the free surface of the parahippocampal gyrus, and was always located within the collateral sulcus (Fig. 3). Area Ph1 reached to most caudal levels among the four areas. It was mainly located in the medial bank of the collateral sulcus, but reached the surface of the parahippocampal gyrus at more rostral levels. Area Ph2 was located more rostrally to Ph1, and was found both in the collateral sulcus and at the free surface of the parahippocampal gyrus. Area Ph3 covered the free surface of the parahippocampal gyrus. The areas differed between each other, and with respect to laterally adjacent areas of the fusiform gyrus by their cytoarchitecture.

### Cytoarchitecture

All areas showed six layers and, therefore, were assigned to the homotypical isocortex. They differed, however, in the width of the layers, transitions between the layers as well as in size, density and arrangement of neurons.

**Fig. 4:**
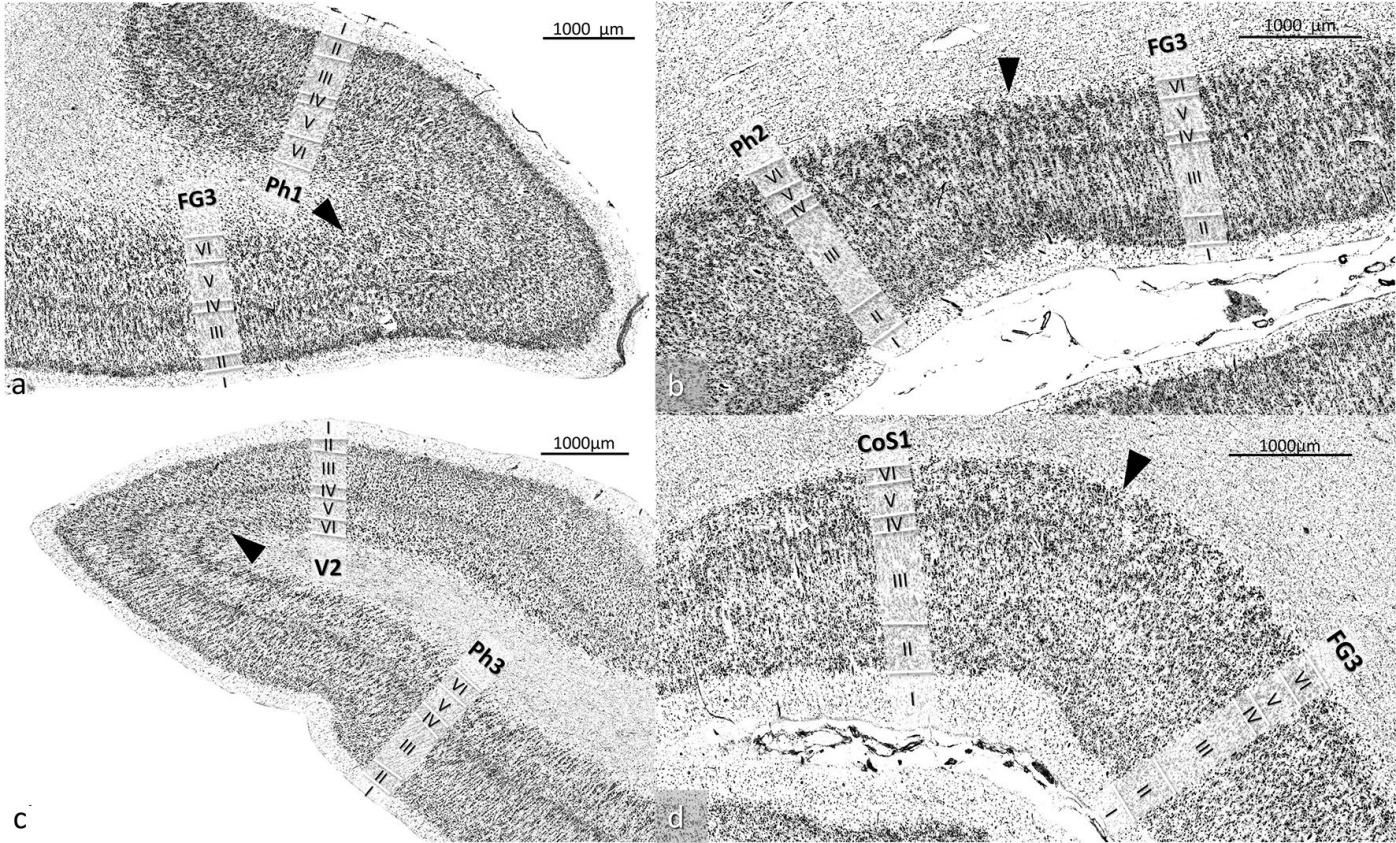
Examples of cytoarchitectonic borders between **a** Ph1 and FG3, **b** Ph2 and FG3, **c** Ph3 and V2 as well as **d** CoS1 and FG3. The histologic images were contrast enhanced for better visualization

**Fig. 5:**
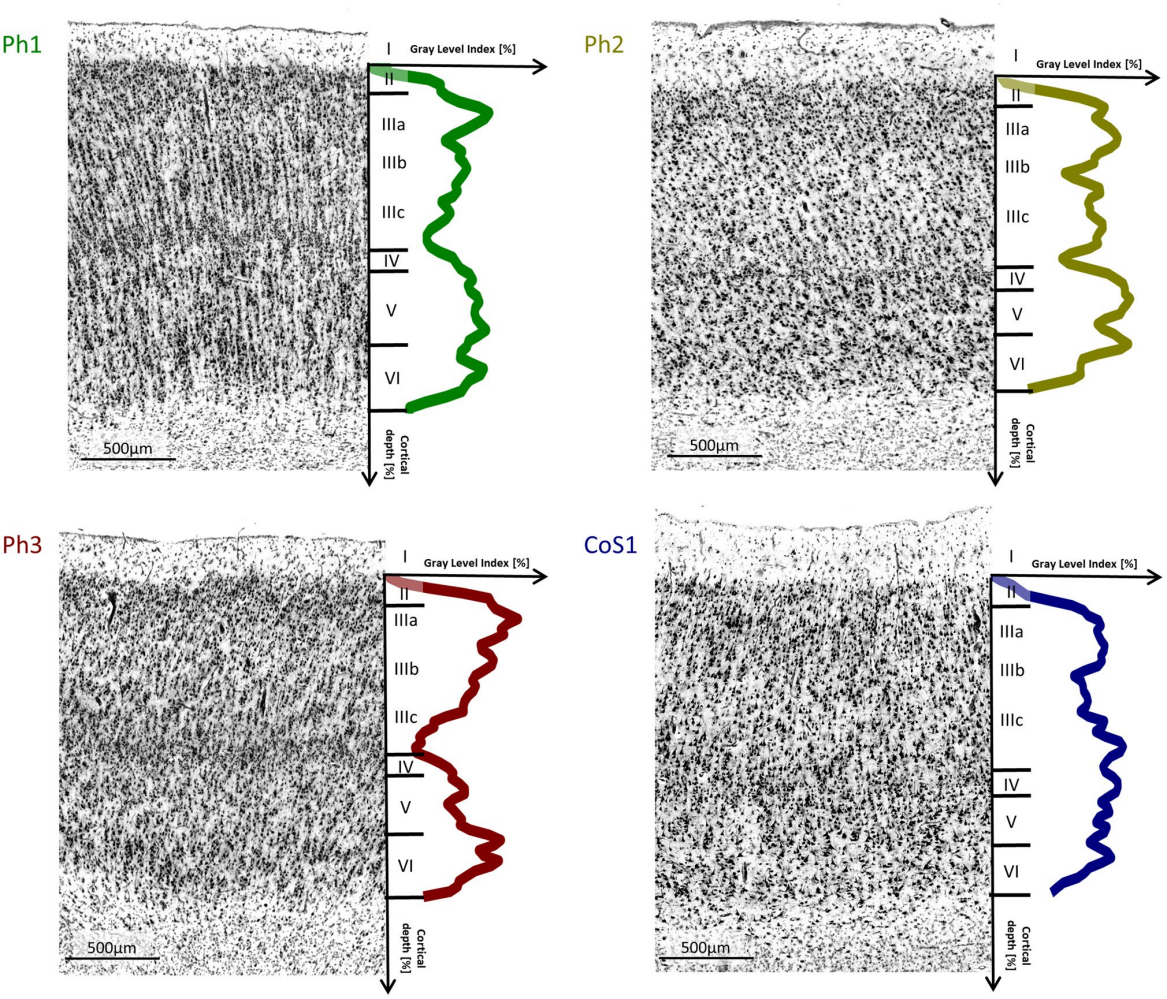
Cytoarchitecture of areas Ph1, Ph2, Ph3 and CoS1. The coloured lines indicate mean GLI profiles. Ph1 was characterized by distinct layers IIIc and VI with predominantly medium-sized pyramidal cells and a clearly visible layer IV. Ph2 was characterized by a layer III with small pyramidal cells and low cell density within all sublayers, a thin and blurred IV and a V with a high density of medium-sized pyramidal cells without a distinct border to VI. Ph3 especially differed from Ph1 by a higher cell density of IV, a layer V with small pyramidal cells and its obvious horizontal stripes due to the high density of IV and VI and the light V in-between them. CoS1 had a thin and light layer IV as well as a slim layer V with a low density of small pyramidal cells. The histologic images were contrast enhanced for better visualization

The caudo-lateral neighbour of area Ph1 was area FG1 at the fusiform gyrus (Caspers et al. 2013) (Fig. 3). Medially, area Ph3 was located. Laterally, area FG3 at the fusiform gyrus (Lorenz et al. 2017), and more rostrally, area Ph2 was found. In contrast to FG3, Area Ph1 showed a less compact, broad and more blurred layer II (Figs. 4a, 5). The transition between layers II and III was fluent. Layer III was subdivided into sublayer IIIa, IIIb and IIIc. The upper parts of layer III contained small pyramidal cells, which increased in size towards the broad sublayer IIIc. This layer showed predominantly medium-sized pyramidal cells with some large pyramidal cells. In contrast to FG3, Ph1 showed more large pyramidal cells in IIIc (Fig. 4a). Layer IV was clearly visible, approximately in the middle of the cortical ribbon. Its cell density was higher than in Ph2 and CoS1, but lower with respect to the cortical cross section than in Ph3. Layer IV was clearly separated from adjacent layers III and V due to its high cell density. Layer V consisted of medium-sized as well as small pyramidal cells and was less cell dense than layer VI. Layer VI showed a high cell packing density of medium-sized pyramidal cells, and its border between cortex and white matter was clear-cut.

Area Ph2 was rostrally adjacent to Ph1 (Fig. 3). In most cases (left hemisphere 9/10, right hemisphere 8/10), area Ph2 was limited medially by the rostral parts of Ph3 before a yet unmapped parahippocampal area became the neighbouring area. This unmapped area is currently part of the gap map “Temporal-to-Parietal” in the Julich-Brain atlas. The rostral boundary was formed by another, yet unmapped area. This area differed from Ph2 by its larger cells in layer V, which showed a soft transition to layer VI. Laterally, Ph2 was adjacent to FG3 at more caudal levels (Lorenz et al. 2017) (Fig. 4b). Rostrally, Ph2 adjoined in most cases laterally to CoS1 (left hemisphere 7/10, right hemisphere 10/10) until a yet unmapped area became the neighbouring area. This unmapped area was characterized by a high density of small cells in layers IIIc and V with a thin, not prominent layer IV in between. The overall cell density of Ph2 was lower than in Ph1 (Fig. 5). Ph2 also showed a rather broad layer II with increasing cell density towards layer III such as found in Ph1. In contrast to the other three areas, Ph2 revealed a rather homogeneous III. Layers IIIa–c contained mainly small pyramidal cells as well as some medium-sized pyramidal cells in IIIc. Layer IV was thin and much less compact than in Ph1 and Ph3. Layers V and VI showed a high density of medium-sized pyramidal cells. The transition between V and VI was fluent, while VI was sharply delineated from the white matter.

Area Ph3 covered parts of the surface of the parahippocampal gyrus (Fig. 3). It was laterally bordered by Ph1 as well as by Ph2 more rostrally. The medial boundary of Ph3 was formed caudally by extrastriate area V2/BA18 (Amunts et al. 2000) (Fig. 4c). Rostrally, V2 was followed by a yet unmapped area. This unmapped area covered parts of the hippocampal fissure and the parahippocampal gyrus and is also part of the gap map “Temporal-to-Parietal” of Julich-Brain. Area Ph3 showed a more compact layer II than Ph1 (Fig. 5). Similar to area Ph1, its layer III was divided into a rather homogeneous sublayer IIIa/IIIb with small pyramidal cells and a sublayer IIIc with medium-sized pyramidal cells. Layer IV showed, compared to the other three areas, the highest cell density and was clearly delimited. Layer V was thinner than in Ph1. It mainly consisted of small pyramidal cells and was clearly separated from layer VI, which predominantly had small pyramidal cells but also some medium-sized pyramidal cells, and an overall higher density than layer V. The laminar pattern of Ph3 was characteristic due to its “horizontal stripes” caused by the high cell density of IV and VI, and the light layer V in-between them. The transition to the white matter was a bit more blurred than in Ph1.

The most rostrally localized area CoS1 was found at the lateral bank of the collateral sulcus (Fig. 3). While it initially bordered on FG3 laterally (Fig. 4d), frontally an unmapped area formed the lateral border. This area delimited CoS1 also rostrally covering the lateral bank of the collateral sulcus. It was characterized by a high density of small cells in layers IIIc and V with a thin and inconspicuous layer IV in between. In most cases (left hemisphere 7/10, right hemisphere 10/10), CoS1 was medially delineated by the cranial parts of Ph2. Then the unmapped area of the gap map “Temporal-to-Parietal” formed the medial border. Area CoS1 showed a light layer II without a distinct border to III (Fig. 5). Layer III was subdivided into sublayers IIIa/IIIb with a high cell density of small and some medium-sized pyramidal cells and sublayer IIIc with lower cell density of small and medium-sized pyramidal cells. Layer III had a higher cell density than layer III of Ph2, but smaller than of Ph1 and Ph3. Layer IV was narrow and appeared light. The borders to layers III and V were also unsharp. A thin layer V predominantly contained small pyramidal cells with a low density. Occasionally, there were medium-sized pyramidal cells. Layer VI was also narrow and consisted of small and medium-sized pyramidal cells, but it had a higher cell density that differentiated it from V. In addition, layer VI was clearly delimited from the white matter.

### Cytoarchitectonic differences and similarities of areas in the PHC and neighbouring fusiform gyrus

**Fig. 6:**
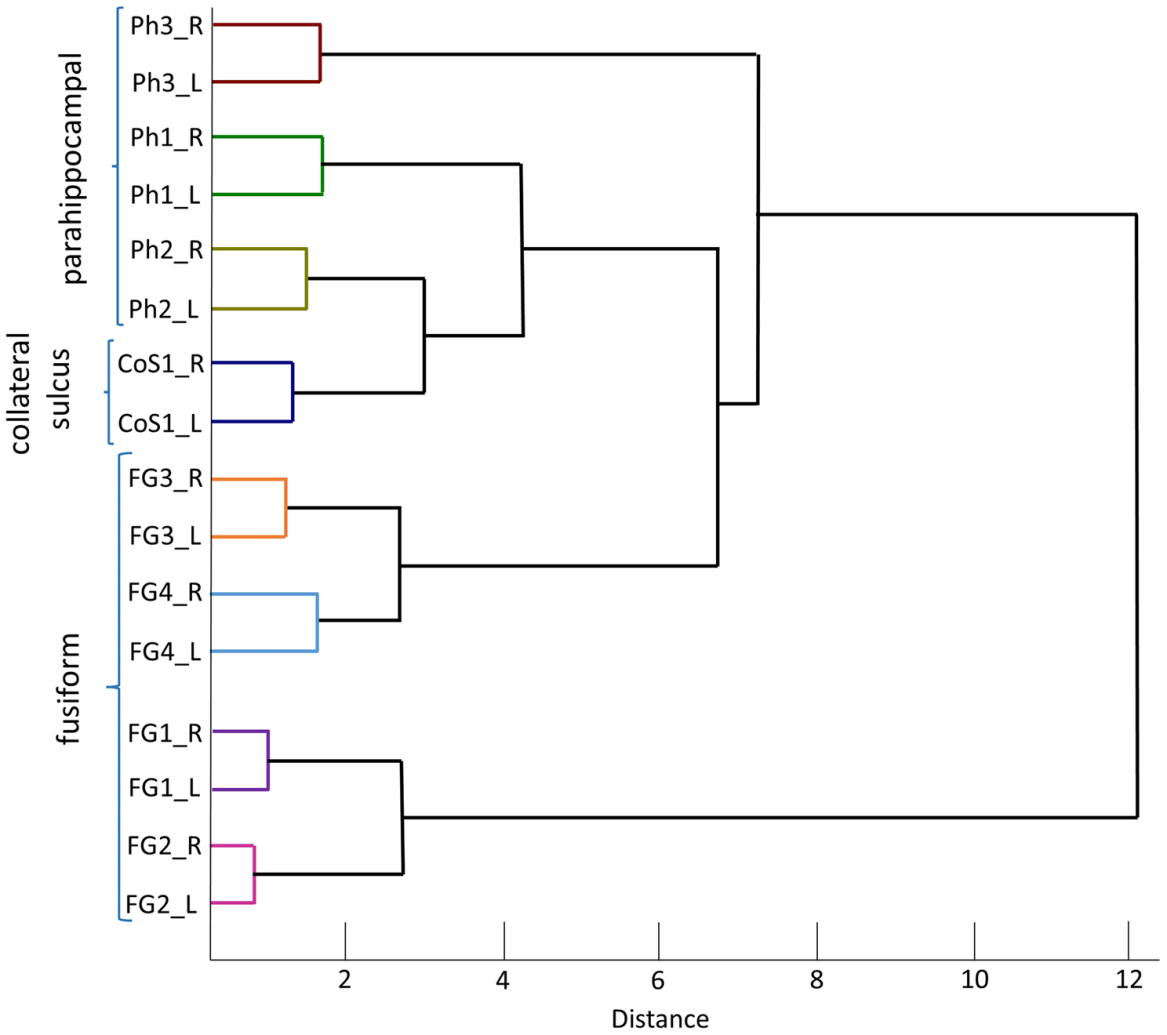
Cluster analysis of the four new parahippocampal areas and adjacent fusiform areas (FG1, FG2, FG3 and FG4). The dendrogram shows a grouping of FG1 and FG2 on one branch, and Ph1, Ph2, Ph3, CoS1 as well as FG3 and FG4 on the second branch. Ph3 is rather separated from the other three parahippocampal areas on grounds of more cytoarchitectonic dissimilarities. FG1 and FG2 are structurally similar to each other and differ from the other six areas (*y*-axis: cytoarchitectonic areas, *x*-axis: Euclidean distance)

The hierarchical cluster analysis of the eight areas Ph1, Ph2, Ph3, CoS1, FG1, FG2, FG3 and FG4 separated fusiform areas FG1 and FG2 from the rest. Here, distances were maximal (Fig. 6). Areas FG3 and FG4 were more similar to the four new areas than FG1 and FG2. The former are more anteriorly located at the fusiform gyrus. Ph3 was most distinct from the other two Ph-areas and area CoS1. Areas Ph2 and CoS1 were more similar to each other than both to Ph1. The smallest distances for each area were found between the two hemispheres.

### Volumes, surface areas and cortical thicknesses of areas

The mean volumes of all four new areas Ph1, Ph2, Ph3 and CoS1 varied between 183.7 mm^3^ and 767.2 mm^3^ (corrected for shrinkage); data are provided in Table 2. Ph1 was the largest area, and CoS1 the smallest. Ph2 and Ph3 were roughly about the same size. Area Ph2 showed the largest standard deviation on the left and Ph1 on the right hemisphere, which showed a trend in terms of interindividual variability. CoS1 revealed the least standard deviation on both sides. The permutation test revealed no significant differences in terms of hemisphere and sex (*p* > 0.05).

**Table 2:** Corrected mean volumes, surface areas as well as cortical thicknesses and corresponding standard deviations (SD) of the four new areas for left and right hemisphere

	Corrected mean volume (mm^3^) ± SD	
	Left hemisphere	Right hemisphere
Area		
Ph1	615.6 ± 288.0	767.2 ± 387.0
Ph2	561.4 ± 323.0	515.0 ± 226.4
Ph3	403.3 ± 278.2	592.3 ± 250.1
CoS1	183.7 ± 59.8	230.9 ± 100.5
Surface area (mm^2^)		
Ph1	261.2	188.6
Ph2	261.8	235.7
Ph3	341.7	149.5
CoS1	47.2	80.4
Cortical thickness (mm) ± SD		
Ph1	2.2 ± 0.8	2.5 ± 0.7
Ph2	2.2 ± 1.2	2.1 ± 0.8
Ph3	2.2 ± 0.8	2.5 ± 0.7
CoS1	1.8 ± 1.2	2.1 ± 0.8

Volumes were calculated as the mean of the ten brains examined, while surface areas and cortical thicknesses were calculated in the MNI-Colin27 reference space

The surface areas of all four new areas Ph1, Ph2, Ph3 and CoS1 varied between 47.2 mm^2^ and 341.7 mm^2^ (Table 2). In accordance to volume, CoS1 also had the smallest surface area on both hemispheres. However, Ph3 had the largest surface area on the left side and Ph2 on the right side.

The cortical thickness showed values between 1.8 mm and 2.5 mm. Again, CoS1 had the smallest value on the left hemisphere, while the other areas had similar cortical thicknesses (Table 2).

### Probability maps and maximum probability map

**Fig. 7:**
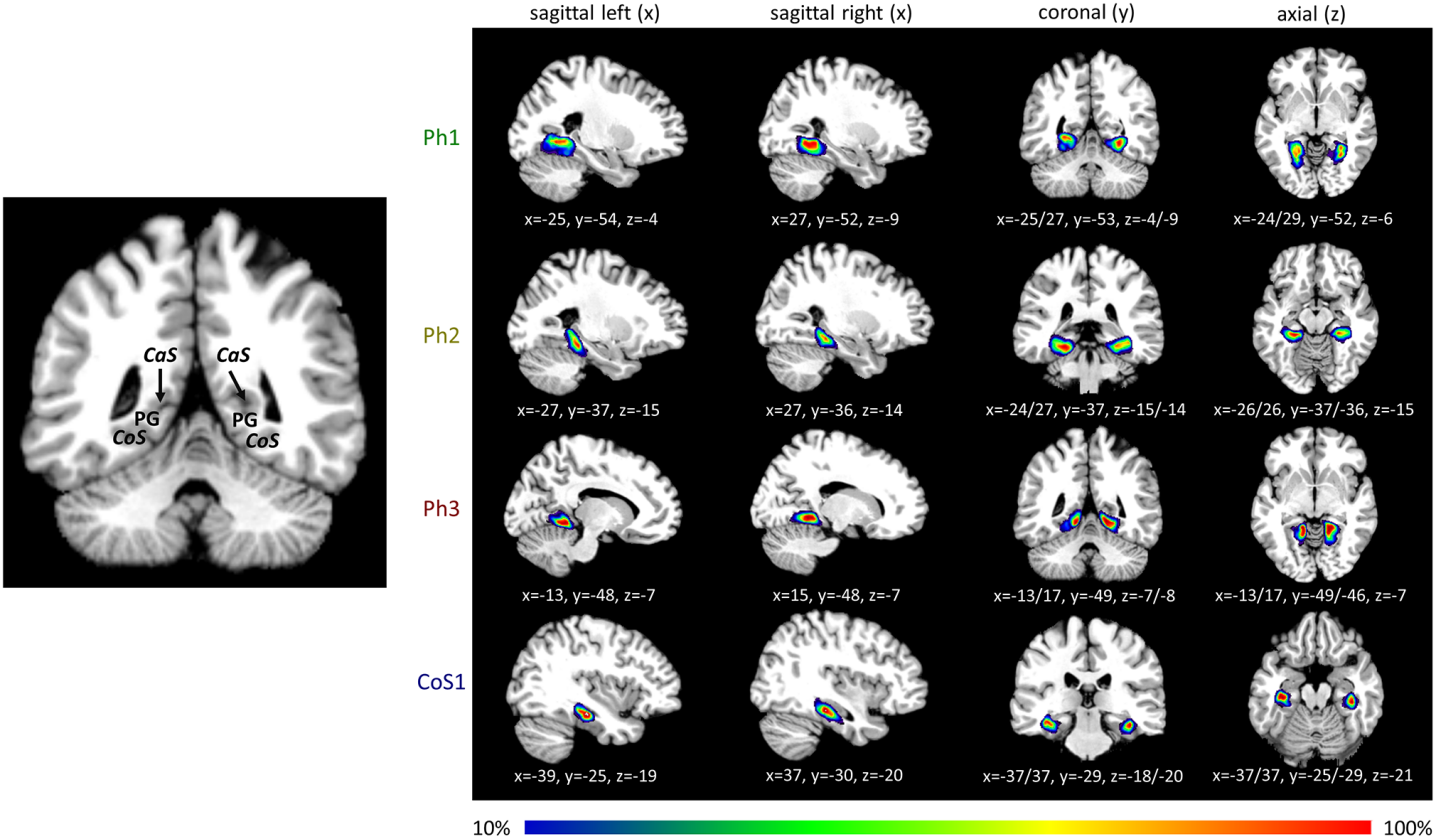
Probability maps of the areas Ph1, Ph2, Ph3 and CoS1 registered to the MNI single subject reference template Colin27. The probabilities are colour-coded. The coordinates are written in white underneath (left/right). A coronal section is shown enlarged displaying the location of the collateral sulcus (CoS), the calcarine sulcus (CaS) as well as the parahippocampal gyrus (PG)

Probability maps of the four new areas were calculated in the two stereotaxic spaces to quantify the intersubject variability in extent and localisation of the areas (Fig. 7). The color-coded maps show regions with a high probability and low intersubject variability in red, while blue indicates a low probability and a high intersubject variability. The centers of gravity of all four areas, left and right hemispheres, in the MNI reference space ICBM152 are listed in Table 3.

**Table 3:** Coordinates of the centers of gravity in MNI ICBM 152 space of probability maps of the four new areas, as well as for comparison, neighbouring fusiform areas FG3 and FG4 for left and right hemisphere

Area	Hemisphere	*x*	*y*	*z*
*Center of gravity coordinates in MNI ICBM 152 space*				
Ph1	Left	−24	−55	−8
	Right	26	−52	−11
Ph2	Left	−29	−40	−14
	Right	30	−38	−14
Ph3	Left	−18	−52	−9
	Right	18	−51	−10
CoS1	Left	−39	−28	−21
	Right	36	−30	−21
FG3	Left	−33	−47	−15
	Right	32	−46	−15
FG4	Left	−45	−48	−18
	Right	43	−45	−20

The map shows that area Ph1 was predominantly located on the medial bank of the collateral sulcus, showing an overlap of the ten brains. The ventral parts showed a less overlap than its center and thus a greater intersubject variability. In general, the localisation of the probability maps of the areas in standard reference space was similar to the localisation of the areas in the individual brains.

**Fig. 8:**
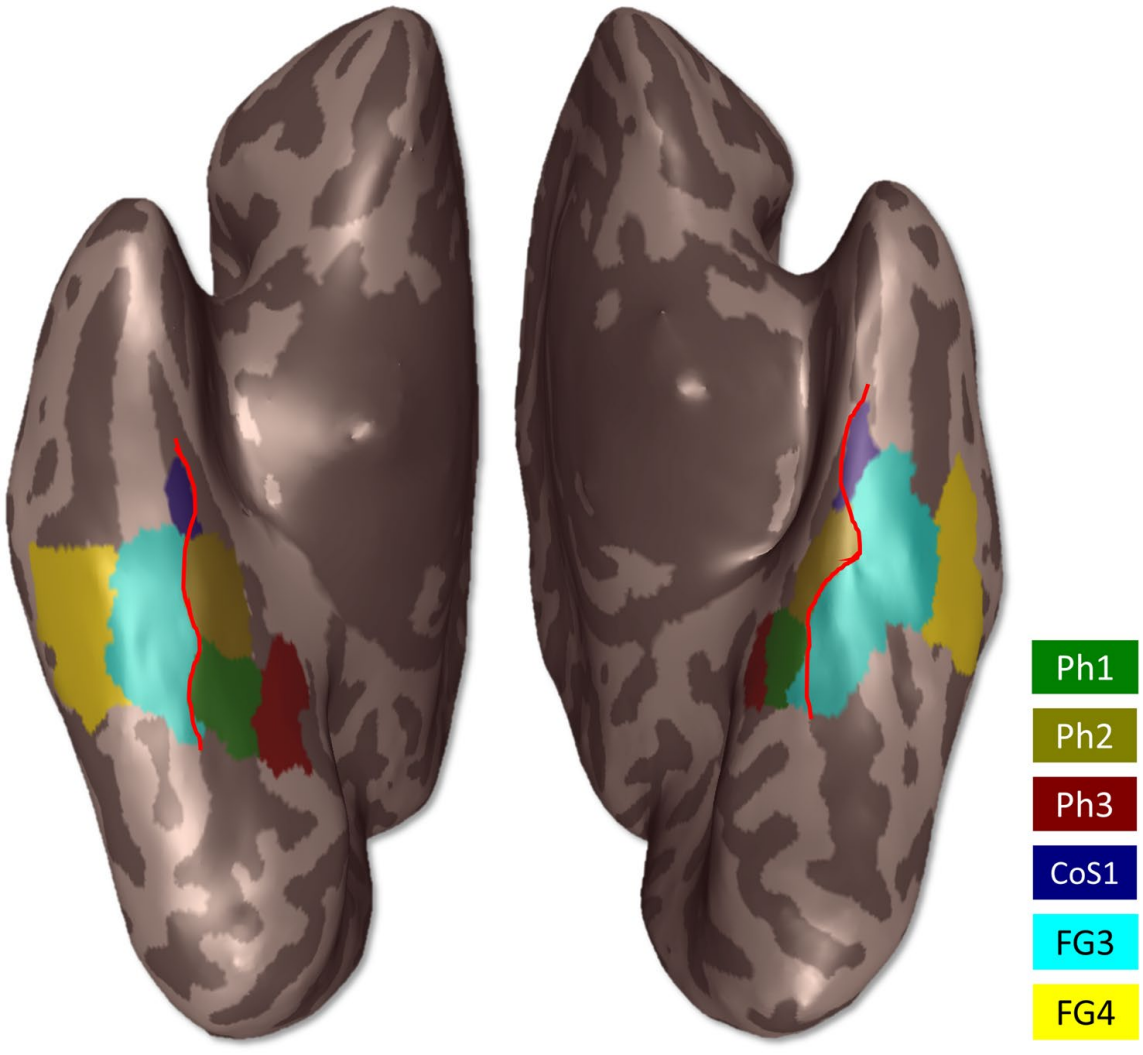
MPM of the four parahippocampal areas registered to the MNI single subject reference template Colin27; inflated view without the cerebellum. Available through https://www.fz-juelich.de/inm/inm-1/julich-brain. Red lines mark the collateral sulcus

The topography of the four new areas and adjacent areas FG3 and FG4 can be seen in the non-overlapping maximum probability map (Fig. 8). Here, Ph1 covered the medial bank of the collateral sulcus at more occipital levels. Ph3 adjoined immediately to Ph1 on its right side covering the surface of the parahippocampal gyrus. At more rostral levels Ph2 bordered on Ph1. Ph2 was also localized on the medial bank of the collateral sulcus. The lateral bank of the collateral sulcus was occupied by FG3, which adjoined laterally to Ph1 as well as Ph2. CoS1 was located frontally from Ph2 and FG3. CoS1 covered the lateral bank of the collateral sulcus.

**Fig. 9:**
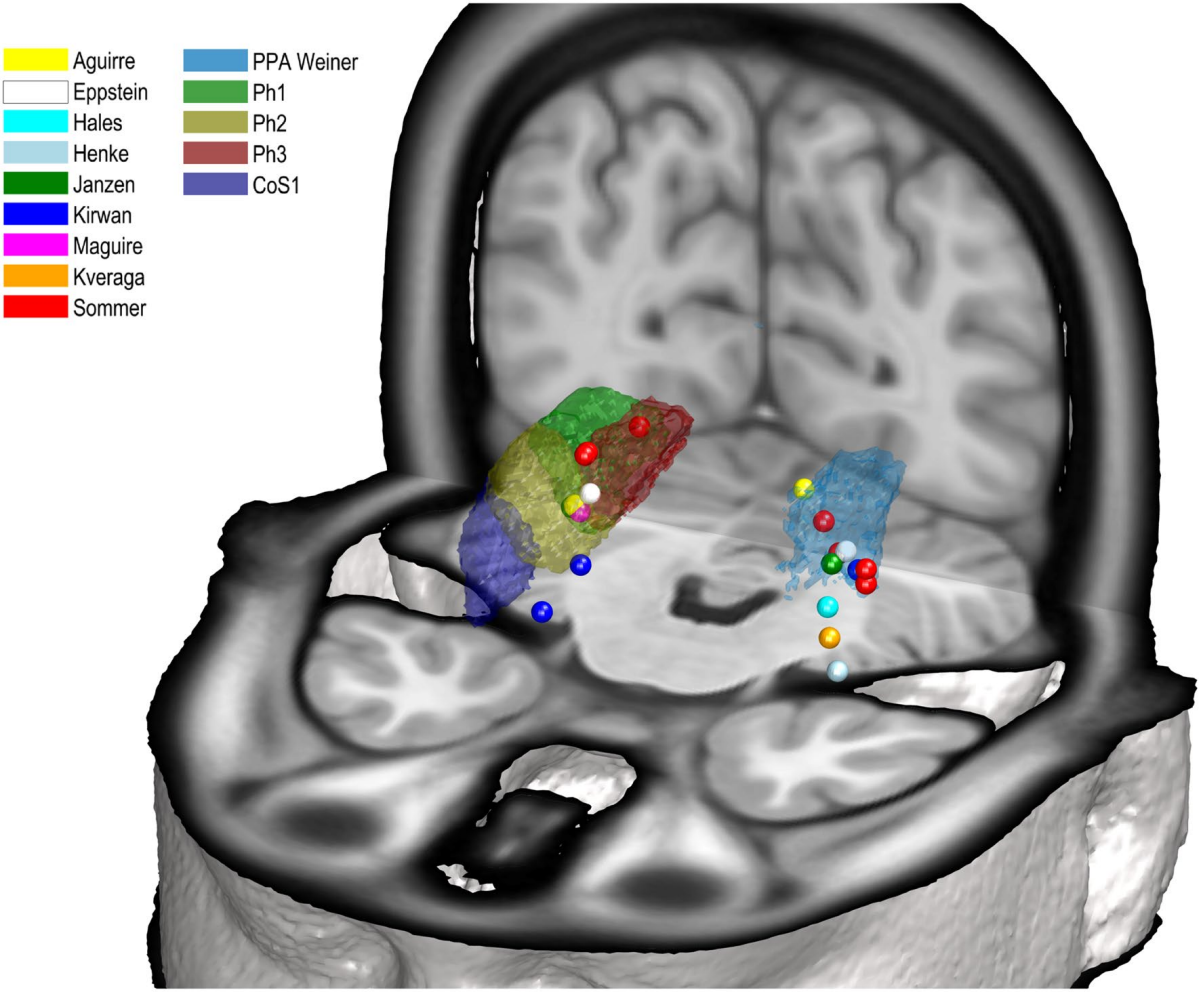
Comparison of cytoarchitectonic maps with positions of functional imaging studies of Aguirre et al. (1996) (yellow), Epstein et al. (1999) (white), Hales et al. (2009) (turquoise), Henke et al. (1999) (grey), Janzen et al. (2007) (green), Kirwan and Stark (2004) (blue), Maguire et al. (1998) (pink), Kveraga et al. (2011) (orange) and Sommer et al. (2005) (red) with surface reconstructions of the MPM of the four new areas in the MNI152 reference space. The dots indicate the coordinates of different activations. The light blue area represents the most probable position of the PPA as described by Weiner et al. (2018). Ph1 is marked in green, Ph2 in yellow, Ph3 in red and CoS1 in blue**.** Coordinates of the named studies are written in Table 4

The superimposition of the MPM with activation peaks from the literature (Table 4), revealed a differentiated distribution of the peaks with respect to the areas of the PHC (Fig. 9), supporting the notion that the four areas are differentially involved in visuo-motor control (more detailed discussion below). All coordinates of the functional imaging data listed in Table 4, converted to native MNI coordinates, are also shown in a sequence of coronal sections as well as in an animation (online resource 1, 2).

**Table 4:** Coordinates of functional imaging studies used for comparison with the localization of the four new areas

Author	Stimuli	*x*	*y*	*z*	Reference space
Aguirre	Topographical learning	23	−40	−7	Talairach
Aguirre	Topographical learning	−15	−52	−3	Talairach
Eppstein	Parahippocampal place area	−28	−39	−6	Talairach
Eppstein	Parahippocampal place area	20	−39	−5	Talairach
Hales	Association of pictures	−26.3	−35.3	−14.8	Talairach
Henke	Association of words	−30	−30	−24	Talairach
Janzen	Association of navigationally relevant objects	−26	−37	−8	Talairach
Janzen	Association of navigationally relevant objects	24	−41	−8	Talairach
Kirwan	Activity for remembered items	25	−29	−22	Approximations of Talairach
Kirwan	Activity for remembered items	−30	−40	−9	Approximations of Talairach
Kirwan	Associative memory retrieval	19	−33	−14	Approximations of Talairach
Kveraga	Objects with strong contextual associations	−30	−30	−26	Average MNI space
Maguire	Environment with salient object	22	−40	−8	Talairach
Sommer	Encoding of object–location associations—object as retrieval cue	21	−63	−9	Standard anatomical space (MNI)
Sommer	Encoding of object–location associations—object as retrieval cue	−36	−33	−15	Standard anatomical space (MNI)
Sommer	Encoding of object–location associations—location as retrieval cue	24	−44	−6	Standard anatomical space (MNI)
Sommer	Encoding of object–location associations—location as retrieval cue	−27	−42	−15	Standard anatomical space (MNI)
Sommer	Common activity pattern for both cue types	24	−45	−6	Standard anatomical space (MNI)
Sommer	Common activity pattern for both cue types	−24	−42	−9	Standard anatomical space (MNI)
Sommer	Confidence of remembering the location associated with a particular object	−36	−33	−12	Standard anatomical space (MNI)

The coordinates are listed in their original described reference space (Aguirre et al. 1996; Epstein et al. 1999; Hales et al. 2009; Henke et al. 1999; Janzen et al. 2007; Kirwan and Stark 2004; Kveraga et al. 2011; Sommer et al. 2005; Maguire et al. 1998)

The maps are available in the Julich-Brain Atlas (https://www.fz-juelich.de/inm/inm-1/julich-brain) and via the “Interactive Atlas Viewer” of the Human Brain Project and the EBRAINS research infrastructure (https://www.humanbrainproject.eu/en/explore-the-brain/atlases/).

## Discussion

The present study revealed four new cytoarchitectonic areas at the caudal parahippocampal gyrus and the collateral sulcus. The comparison with functional imaging data suggests that they also differ in terms of their role in visual–spatial orientation as well as encoding associative memories. The cytoarchitectonic segregation of the caudal parahippocampal gyrus and the collateral sulcus may provide a microstructural correlate of the functional heterogeneity and complexity of this region that has been described in the literature. Moreover, stereotaxic maps were provided as probability maps, i.e., they captured intersubject variability at each position in space. The variability, however, was less high than in other associative areas of previous studies of our group, e.g., Broca’s region. Moreover, the relationships of the areas with respect to neighbouring sulci was rather stable.

The four areas differed in cytoarchitecture, which was quantified by cluster trees, measuring their similarities and dissimilarities as Euclidian distances. The cytoarchitecture of areas Ph2 and CoS1 was most similar as compared to all others: both revealed a light layer II without a clear-cut border to III, a low cell density in layer III and a thin and cell-poor layer IV. The two areas are located further rostrally than the other two areas. These similarities resulted in a small distance between the two areas in the cluster analysis suggesting also a functional similarity of these areas. Area Ph1 was characterized by a higher density of larger pyramidal cells than Ph2 and CoS1 that were especially found in layer IIIc, layer V as well as in layer VI. This difference in structure was also represented by a greater distance between Ph1 and Ph2/CoS1 in the cluster analysis. Area Ph3 differed from the other three by clear horizontal stripes resulted of a high cell density of layer II, IV and VI with lighter layers III and V in between. Ph3 showed the greatest distance to the other three areas meaning suggesting functional differences.

### Comparison with previous architectonic maps

With respect to Brodmann’s cytoarchitectonic map (Brodmann 1909), the four new areas were localized within the caudal part of his area 28. Brodmann has described BA28 as an area with a high cell density, medially adjacent to the rhinal sulcus. He did not further subdivide this area, and did not show the cytoarchitecture within the sulcus. In contrast, we identified four areas in this region, including one area in the depth of the collateral sulcus.

Areas Ph1, Ph2, Ph3 and CoS1 seem to cover areas TH as well as partly PH of von Economo and Koskinas (1925) (Fig. 1). In correspondence to the localisation of areas Ph1 and Ph3 of the present study, von Economo described the medially located parts of area PH in the posterior collateral sulcus and parahippocampal gyrus. Area PH was described as inhomogeneous depending on the neighbouring areas from which PH took on individual characteristics. Overall, area PH had clear and broad layers II and IV. While layer IV had a high density, V consisted of small cells. The boundary between the cortical ribbon and white matter was distinct. Matching Ph1, subtype PHT (temporal) was characterized by a layer III with occasional large cells in IIIc and a clear distinction of V and VI. Ph3 seems to correspond to subtype PHO (occipital) with occasional large cells in IIIc, while cells of layer IV were smaller than in PHT. The delimitation between layers V and VI was sharp. Similar to TH, area CoS1 was found in the depth of the collateral sulcus, while Ph2 was located at the medial bank of the collateral sulcus. The description of an indistinct layer II and a low cell density of layer III by von Economo and Koskinas was also consistent with the cytoarchitecture of Ph2 and CoS1. Similar to Ph2, layer V showed a higher density of larger pyramidal cells, and layer VI also had a high cell density. The border between the cortex and the white matter was distinct. In summary, TH was consistent with Ph2 and CoS1 in both localisation and cytoarchitecture.

It is difficult to go beyond this point and discuss in more detail correspondence between different maps, since an important added value of the probabilistic maps is the inclusion of the variability between brains. Any historical brain map, such as that from von Economo and Koskinas, based on a selection of sections of one or a few individual brains cannot cover this aspect, and it is not possible to compare them with sufficient accuracy in a common reference space.

### Comparison with neuroimaging studies

Compared to the representation of the examined region in previous anatomical maps, the functional segregation of this region is much more heterogeneous and more fine-grained subdivisions have been proposed. Several studied demonstrated that the PHC participates in visual–spatial tasks including map reading, spatial orientation, navigation and spatial memory (Epstein and Kanwisher 1998; Aguirre et al. 1996; Janzen et al. 2007; Maguire et al. 1998; Aguirre and D'Esposito 1999; Mellet et al. 2000). The coordinates of area PPA as identified by Epsteins and colleagues (1999) mainly overlapped with Ph2 in both hemispheres (Fig. 9). Aguirre et al. (1996) coordinates for topographical learning were localized in area Ph2 on the right and area Ph3 on the left side (Fig. 9). Janzen et al. (2007) reported coordinates of increased activity in the recognition of objects that had previously been placed at decision points of mazes and thus served as landmarks, which mainly matched area Ph2 in both hemispheres (Fig. 9). Another study described increased activity during exploration of an environment that contained salient objects as well as textures (Maguire et al. 1998), which matched the transition zone between Ph1, Ph2 and Ph3 of the right hemisphere (Fig. 9). Sommer et al. (2005) showed activities elicited by encoding of object–location associations, while coordinates matched the localisation of the parahippocampal areas: while objects as retrieval cues elicited activity within the transition between Ph1 and Ph3 in the right hemisphere, activity in the left hemisphere was mainly localized within CoS1. Activity for location as retrieval cue was localized within the transition between area Ph1 and Ph3 on the right side and within area Ph2 on the left side. Common activity pattern for both cue types was found in the transition between Ph1 and Ph3 right as well as in the transition between Ph1 and Ph2 left. Activity elicited by remembering the location associated with a particular object revealed a coordinate that was located within the transition between CoS1 and Ph2 in the left hemisphere (Fig. 9).

Furthermore, the PHC is involved in formation of episodic memory (Davachi et al. 2003; Kirwan and Stark 2004; Tendolkar et al. 2008; Düzel et al. 2003; Henke et al. 1999; Hales et al. 2009; Yang et al. 2008). Henke et al. (1999) provided coordinates of an area exhibiting great activity elicited by association of words that was localized at the height of CoS1 without matching it (Fig. 9). Kveraga reported coordinates for activity caused by objects with strong contextual associations that were located close to CoS1 (Kveraga et al. 2011) (Fig. 9). In addition, increased activity during associative encoding of pairs of images was localized within Ph2 (Hales et al. 2009) (Fig. 9). Furthermore, coordinates of increased activity for associative memory during encoding and retrieval of face–name associations were located at the height of CoS1 without matching it on the right side, within Ph2 on the left side and at the height of the right transition zone between Ph2 and CoS1 without matching them (Kirwan and Stark 2004) (Fig. 9). The coordinates of these studies are listed in Table 4.

Most recently, Rosenke et al. (2021) published a map of early visual and category-selective regions in human ventral and lateral occipito-temporal cortex. A comparison of this map with our brain maps revealed a match of the area mFus-faces, which corresponded to the FFA-2, with CoS1. In addition, there was a match of area CoS-places, which corresponded to the PPA, with our areas Ph1, Ph2 and Ph3.

Thus, functional imaging data result in a detailed pattern of segregation pf the PHC: while the anterior part of the PHC seems to be activated by nonspatial associations, activity in the posterior part is elicited by stimuli associated with spatial contexts (Aminoff et al. 2007; Bar and Aminoff 2003; Baumann and Mattingley 2016). The present results provide further evidence for a subdivision into an anterior and a posterior part. In line with this, the discriminant analysis of our four new areas also resulted in a grouping of the rostrally localized areas CoS1 and Ph2 on the one hand, and the caudal located areas Ph1 and Ph3 on the other. Moreover, the comparison with functional imaging data showed activations elicited by associations within the rostral areas. However, activations elicited by visual–spatial information were found in the caudal areas. Interestingly, Weiner et al. provided data about the most probable location of the parahippocampal place area (Weiner et al. 2018), that partly covered area Ph1, Ph2 as well as Ph3 (Fig. 9). This leads to the hypothesis that the PPA can also be further subdivided. Appropriately, it was demonstrated by Baldassano, Beck, and Fei-Fei that the connections of the PPA also have an anterior–posterior subdivision: while the posterior part had stronger connections to visual associated regions, the anterior part was more strongly connected to the parietal and retrosplenial cortex (Baldassano et al. 2013).

Some stereotaxic coordinates of studies from the literature didn’t match exactly the new four areas, although functionally, there was an overlap with activation data. Such mismatch may have different reasons. For example, filtering of functional imaging data with a radius of 5 mm and more may lead to such divergence. Second, different reference spaces were used and terms such as “MNI space” were not always clearly defined. Though, depending on the position of the line between anterior and posterior commissure, the position of stereotactic coordinates may vary. This makes it difficult to assess the correlation of cytoarchitectonic areas with functional imaging data solely by comparing their stereotaxic coordinates. Anatomical precision can be added, for example, using the same workflows to align both anatomical and functional data into the same reference space, and/or using surface-based alignments, which seems to reduce intersubject variability, e.g., Fischl et al., Rosenke et al.. This remains to be a project of future research.

In conclusion, the present study identified four new cytoarchitectonic areas in the parahippocampal gyrus and the collateral sulcus, and provided probability maps in stereotaxic spaces. These maps represent a further step towards a higher coverage of the cortex in this functionally so important region. They can be used as an anatomical reference to relate data of other modalities and functional neuroimaging and connectivity studies to this region, and could be a tool to further sharpen our concepts on visuo-spatial processing and episodic memory.

## Supplementary Information

Below is the link to the electronic supplementary material.

Supplementary file1: Coordinates of functional imaging studies of Aguirre et al. (1996) (yellow), Epstein et al. (1999) (white), Hales et al. (2009) (turquoise), Henke et al. (1999) (grey), Janzen et al. (2007) (green), Kirwan and Stark (2004) (blue), Maguire et al. (1998) (pink), Kveraga et al. (2011) (orange) and Sommer et al. (2005) (red) converted to native MNI coordinates and shown as coloured dots in a sequence of coronal sections within the MNI152 reference space. The corresponding native MNI coordinates are written above the respective image. Coordinates of the named studies are also written in Table 4 (PNG 1815 KB)

Supplementary file2: Comparison of cytoarchitectonic maps with positions of functional imaging studies of Aguirre et al. (1996) (yellow), Epstein et al. (1999) (white), Hales et al. (2009) (turquoise), Henke et al. (1999) (grey), Janzen et al. (2007) (green), Kirwan and Stark (2004) (blue), Maguire et al. (1998) (pink), Kveraga et al. (2011) (orange) and Sommer et al. (2005) (red) with surface reconstructions of the MPM of the four new areas in the MNI152 reference space. The dots indicate the coordinates of different activations. The light blue area represents the most probable position of the PPA as described by Weiner et al. (2018). Ph1 is marked in green, Ph2 in yellow, Ph3 in red and CoS1 in blue. Coordinates of the named studies are written in Table 4 (MP4 9490 KB)

## Data Availability

All maps are available in the Julich-Brain Atlas (https://www.fz-juelich.de/inm/inm-1/julich-brain) and via the EBRAINS platform of the Human Brain Project (https://ebrains.eu/service/human-brain-atlas/).

## Code availability

Amunts et al. (2020) published the complete methodological procedure and various code repositories.
