# Supplementary figures and images for: Cytoarchitectonic parcellation and functional characterization of four new areas in the caudal parahippocampal cortex
Source: Brain Struct Funct. 2022 Jan 6;227(4):1439–55. doi: 10.1007/s00429-021-02441-2 (PMC9046293; doi:10.1007/s00429-021-02441-2)

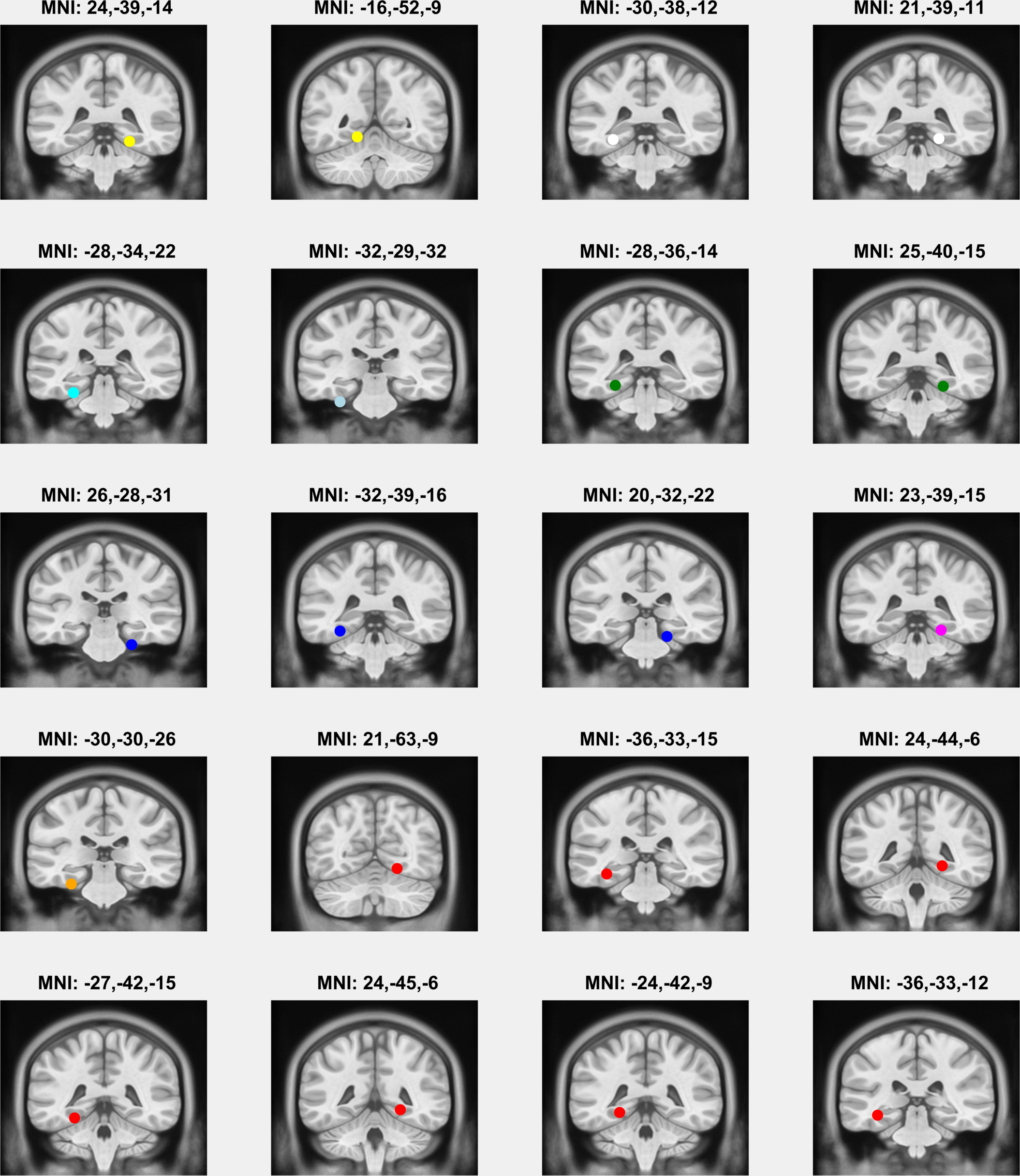

Supplement: Supplementary file 1 — Supplementary file1: Coordinates of functional imaging studies of Aguirre et al. (1996) (yellow), Epstein et al. (1999) (white), Hales et al. (2009) (turquoise), Henke et al. (1999) (grey), Janzen et al. (2007) (green), Kirwan and Stark (2004) (blue), Maguire et al. (1998) (pink), Kveraga et al. (2011) (orange) and Sommer et al. (2005) (red) converted to native MNI coordinates and shown as coloured dots in a sequence of coronal sections within the MNI152 reference space. The corresponding native MNI coordinates are written above the respective image. Coordinates of the named studies are also written in Table 4 (PNG 1815 KB) [file 429_2021_2441_MOESM1_ESM.png]
